# Complete genomes of *Escherichia coli* isolates encoding the EatA autotransporter from pediatric sources

**DOI:** 10.1128/mra.01127-25

**Published:** 2026-03-23

**Authors:** Bernadette A. Hritzo, Jane M. Michalski Wilhelm, David A. Rasko

**Affiliations:** 1 Institute for Genome Sciences, University of Maryland School of Medicine, Baltimore, Maryland, USA; 2 Department of Microbiology and Immunology, University of Maryland School of Medicine 12264, Baltimore, Maryland, USA; 3 Department of Microbial Pathogenesis, University of Maryland School of Dentistry 89015 , Baltimore, Maryland, USA; 4 Center for Pathogen Research, University of Maryland School of Medicine 12264, Baltimore, Maryland, USA; Loyola University Chicago, Chicago, Illinois, USA

**Keywords:** *Escherichia coli*, ETEC, virulence, EatA, plasmid, genome

## Abstract

Virulence in *Escherichia coli* is multifactorial, with additional virulence factors continuously being examined and characterized. We describe the complete genomes of 12 *E. coli* isolates obtained from subjects enrolled in the Global Enteric Multicenter Study, each containing the virulence-associated enterotoxigenic *E. coli* autotransporter A gene (*eatA*).

## ANNOUNCEMENT

While not considered a canonical virulence gene of enterotoxigenic *Escherichia coli* (ETEC), there is mounting evidence that the ETEC autotransporter A (*eatA*) plays a critical role in ETEC virulence (1). We describe the complete genomes of 12 *E. coli* isolates (2) containing the *eatA* gene (1, 3) from each of the seven geographic sites of the Global Enteric Multicenter Study (4). Phylogenomically diverse *eatA*-containing isolates from cases and controls, as well as male and female subjects, were selected for complete genome sequencing (Table 1).

**TABLE 1:** Isolate and genome metrics

Isolate information								Sequencing statistics											Accession numbers			
Isolate	Country	Case/control	Sex	Year of isolation	Phylogenetic group	Serotype	ST	Molecule name	Size (bp)	Illumina read pairs^*a*^	Illumina bases^*a*^	ONT trimmed reads^*a*^	ONT trimmed bases^*a*^	ONT N50	Genome coverage^*a*^	No. of contigs	Total genome length (bp)	GC (%)	BioProject	Assembly	SRA (Illumina)	SRA (ONT)
100117	Gambia	Case	Female	2008	Cryptic	OND:H45	485	E100117	5,053,006	3,198,198	983,510,344	157,308	513,659,526	3,299.5	273.3	6	5,478,516	50.25	PRJNA1321367	JBRERF010000001	SRR35329244	SRR35329229
								pE100117_127	127,487											JBRERF010000002		
								pE100117_123	123,295											JBRERF010000003		
								pE100117_121^*b*^	121,255											JBRERF010000004		
								pE100117_50	50,722											JBRERF010000005		
								pE100117_3	2,751											JBRERF010000006		
100341	Gambia	Case	Female	2008	A	O7:H4	484 (168)	E100341	4,860,512	3,530,593	1,086,152,515	179,773	762,931,855	4,265.0	357.5	5	5,172,020	50.53	PRJNA1322554	JBRERQ010000001	SRR35332983	SRR35332980
								pE100341_124^*b*^	123,858											JBRERQ010000002		
								pE100341_119	119,093											JBRERQ010000003		
								pE100341_66	66,469											JBRERQ010000004		
								pE100341_2	2,088											JBRERQ010000005		
200086	Mali	Control	Male	2008	Cryptic I	OND:H45	485	E200086	5,179,901	3,063,393	939,867,671	612,423	1,861,035,025	3,057.0	489.1	9	5,727,105	50.28	PRJNA1322554	JBRERP010000001	SRR35332982	SRR35332979
								pE200086_121^*b*^	121,281											JBRERP010000002		
								pE200086_120	120,396											JBRERP010000003		
								pE200086_110	110,583											JBRERP010000004		
								pE200086_79	77,979											JBRERP010000005		
								pE200086_59	59,766											JBRERP010000006		
								pE200086_49	49,559											JBRERP010000007		
								pE200086_59	4,885											JBRERP010000008		
								pE200086_3	2,755											JBRERP010000009		
200139	Mali	Case	Female	2008	B1	O104:H2	678	E200139	4,932,310	3,362,912	1,034,895,605	1,126,528	1,325,135,994	1,204.3	442.0	7	5,339,051	50.66	PRJNA1322554	JBRERO010000001	SRR35332971	SRR35332978
								pE200139_148	148,227											JBRERO010000002		
								pE200139_112^*b*^	112,455											JBRERO010000003		
								pE200139_78	77,987											JBRERO010000004		
								pE200139_60	60,142											JBRERO010000005		
								pE200139_5	4,686											JBRERO010000006		
								pE200139_3_2	3,244											JBRERO010000007		
203449	Mali	Control	Female	2009	A	O8:H10	5386	E203449	4,985,798	3,738,736	1,151,163,899	451,976	687,167,023	1,546.2	348.2	6	5,280,152	50.63	PRJNA1322554	JBRERN010000001	SRR35332968	SRR35332977
								pE203449_121	121,928											JBRERN010000002		
								pE203449_106^*b*^	106,280											JBRERN010000003		
								pE203449_57	57,717											JBRERN010000004		
								pE203449_4_2	4,232											JBRERN010000005		
								pE203449_4_1	4,197											JBRERN010000006		
300010	Mozambique	Control	Female	2007	D	O23:H15	70	E300010	5,055,009	4,002,638	1,233,426,158	239,953	439,745,417	1,852.5	309.4	9	5,408,625	50.48	PRJNA1322554	JBRERM010000001	SRR35332967	SRR35332976
								pE300010_105	105,941											JBRERM010000002		
								pE300010_86	86,575											JBRERM010000003		
								pE300010_79	79,468											JBRERM010000004		
								pE300010_68^*b*^	68,198											JBRERM010000005		
								pE300010_5	5,271											JBRERM010000006		
								pE300010_4	4,510											JBRERM010000007		
								pE300010_2	2,101											JBRERM010000008		
								pE300010_1	1,552											JBRERM010000009		
300082	Mozambique	Case	Male	2008	B1	O91:H10	12313	E300082	4,572,448	3,688,114	1,134,203,909	145,832	489,282,805	3,401.0	311.9	7	5,205,800	50.56	PRJNA1322554	JBRERL010000001	SRR35332966	SRR35332975
								pE300082_261	261,732											JBRERL010000002		
								pE300082_153^*b*^	153,406											JBRERL010000003		
								pE300082_104	104,543											JBRERL010000004		
								pE300082_56	56,826											JBRERL010000005		
								pE300082_50	50,961											JBRERL010000006		
								pE300082_6	5,884											JBRERL010000007		
300446	Mozambique	Control	Female	2008	Cryptic I	OND:H45	485	E300446	5,019,886	3,356,876	1,030,449,026	268,043	821,078,065	3,070.8	348.6	7	5,311,476	50.29	PRJNA1322554	JBRERK000000001	SRR35332965	SRR35332974
								pE300446_128	128,494											JBRERK010000002		
								pE300446_81^*b*^	81,849											JBRERK010000003		
								pE300446_72	72,150											JBRERK010000004		
								pE300446_5	5,099											JBRERK010000005		
								pE300446_3	2,757											JBRERK010000006		
								pE300446_1	1,241											JBRERK010000007		
400634	Kenya	Case	Female	2008	Cryptic I	OND:H45	485	E400634	5,038,625	3,906,639	1,201,154,153	246,927	660,754,750	2,716.9	349.7	7	5,323,749	50.15	PRJNA1322554	JBRERJ010000001	SRR35332964	SRR35332973
								pE400634_103	103,452											JBRERJ010000002		
								pE400634_87^*b*^	87,445											JBRERJ010000003		
								pE400634_48	48,180											JBRERJ010000004		
								pE400634_33	32,917											JBRERJ010000005		
								pE400634_8	7,952											JBRERJ010000006		
								pE400634_5	5,178											JBRERJ010000007		
500002	India	Case	Male	2007	Cryptic I	OND:H45	485	E500002	4,973,609	3,538,598	1,085,172,960	226,565	501,934,622	2,246.4	301.4	6	5,266,097	50.2	PRJNA1322554	JBRERI010000001	SRR35332963	SRR35332972
								pE500002_123^*b*^	123,337											JBRERI010000002		
								pE500002_112	112,330											JBRERI010000003		
								pE500002_49	49,554											JBRERI010000004		
								pE500002_5	5,178											JBRERI010000005		
								pE500002_2	2,089											JBRERI010000006		
600003	Bangladesh	Case	Male	2007	Cryptic I	OND:H45	485	E600003	4,933,499	2,785,935	840,338,429	203,157	486,787,717	2,431.0	257.5	6	5,154,834	50.17	PRJNA1322554	JBRERH010000001	SRR35332962	SRR35332970
								pE600003_100	100,740											JBRERH010000002		
								pE600003_68^*b*^	67,796											JBRERH010000003		
								pE600003_47	46,687											JBRERH010000004		
								pE600003_4	4,011											JBRERH010000005		
								pE600003_2	2,101											JBRERH010000006		
700025	Pakistan	Control	Male	2008	D	O166:H15	349 (349)	E700025	4,964,031	3,633,291	1,115,663,409	145,654	338,604,055	2,375.5	283.5	5	5,129,814	50.63	PRJNA1322554	JBRERG010000001	SRR35332981	SRR35332969
								pE700025_154^*b*^	154,569											JBRERG010000002		
								pE700025_5_7	5,775											JBRERG010000003		
								pE700025_4	3,887											JBRERG010000004		
								pE700025_1_5	1,552											JBRERG010000005		

^*a*^ Values refer to the complete genome project.

^*b*^ Gray highlight indicates *eatA-*containing plasmid.

Initial isolation is described by Panchalingam et al. (5). Briefly, feces or rectal swabs (transported in cold containers) were inoculated into Cary–Blair transport media then selective growth media within 6 and 18 hours of collection. Several lactose-fermenting colonies (red/pink) were selected from McConkey agar (incubated for 48 hours at 37°C) and sub-cultured in motility indole ornithine medium. Suspected *E. coli* isolates [motility(−), indole ornithine(−), methyl-red(+), Voges–Proskauer(−), citrate(−)] were lacking canonical pathotype genes as determined by multiplex PCR (6). Verified *E. coli* isolates were frozen in 20% glycerol and maintained at −80°C. Frozen cultures were provided to, and stored at, the Center for Vaccine Development, University of Maryland School of Medicine.

Isolates were resurrected from frozen culture on lysogeny agar overnight at 37°C. Genomic DNA was extracted using Qiagen’s DNeasy Blood & Tissue Kit, and sequencing libraries for both sequencing technologies were generated with unsheared genomic DNA. Nanopore libraries were prepared using Oxford Nanopore Technologies’ (ONT) Genomic DNA by Ligation Kit (7) and run on Nanopore R9 flow cells with the MinION Mk1B sequencer. Basecalling was performed using Guppy (v4.2.2) (8). Illumina libraries (150 bp paired end) were prepared using the Kapa Library Kit (Roche/Illumina) and sequenced on the Illumina HiSeq 4000 platform. Quality control and adapter trimming were performed with bcl2fastq (v2.20.0.445) (9) and porechop (v0.2.3_seqan2.1.1) (8) for Illumina and ONT sequencing, respectively. Hybrid assembly using both Illumina and ONT reads was conducted with Unicycler (v0.4.8) (10). Final assembly statistics were recorded with QUAST (5.0.2) (11). Genomes were annotated with the NCBI Prokaryotic Genome Annotation Pipeline (v6.10) (12). All software was run with default values.

Isolate information, relevant sequencing statistics, and GenBank accession numbers are included in Table 1. Average genome coverage was 339.3× (range 257–489×), and the assembled genomes have an average genome size of 5,316,437 bp (range 5,129,814–5,727,105 bp) and an average GC% of 50.4% (range 50.15%–50.7%). Analysis of these 12 *eatA-*positive isolates determined the sequence type (MLST) (13, 14) and molecular serotype (Serotype Finder v1.1 [15, 16]). Together, these isolates represent seven distinct sequence types and serotypes (Table 1).

The plasmid-encoded *eatA* gene was harbored on plasmids ranging from 68 to 153 kb, indicating *eatA* is carried on diverse plasmids and is phylogenomically distributed.

## Data Availability

All data have been released in BioProjects PRJNA1321367 and PRJNA1322554. The associated SRA and genome assembly accession numbers for each isolate are listed in Table 1.
